# *Pyrenophora tritici–repentis* in Tunisia: Race Structure and Effector Genes

**DOI:** 10.3389/fpls.2019.01562

**Published:** 2019-12-18

**Authors:** Sana Kamel, Mejda Cherif, Mohamed Hafez, Therese Despins, Reem Aboukhaddour

**Affiliations:** ^1^Laboratory of Genetics and Cereal Breeding, Department of Agronomy and Plant Biotechnology, National Agronomic Institute of Tunisia, University of Carthage, Tunis, Tunisia; ^2^Cereal Pathology Lab, Agriculture and Agri-Food Canada, Lethbridge Research and Development Centre, Lethbridge, AB, Canada

**Keywords:** *Pyrenophora tritici–repentis*, necrotrophic effectors, *ToxA*, *ToxB*, haplotypes, Tunisia

## Abstract

Tan spot is a destructive foliar wheat disease worldwide and caused by the ascomycete fungus *Pyrenophora tritici–repentis* (*Ptr*); it has become more frequent in Tunisia over the last decade. In this study, the virulence of 73 single-spore isolates, collected from durum and bread wheat fields during 2017–2018 growing season, was evaluated on four differential wheat genotypes. This was followed by polymerase chain reaction tests with specific primers for the effector genes *ToxA*, *ToxB*, and *toxb* (*ToxB*-homolog). Sequence analysis to validate the identity of the amplified genes was followed, and *ToxA* amplicons from a subset of 22 isolates were analyzed to determine its haplotype identity. *Ptr* isolates from Tunisia were grouped in races 2, 4, 5, and 7, and 44% of the tested isolates did not fit under any known race, and were denoted here as atypical. These atypical isolates induced the same symptoms as race 7 isolates, extensive necrosis, and chlorosis on susceptible genotypes, but lacked the *ToxA* gene. *ToxA* is the only identified necrosis-inducing effector in *Ptr*, and was amplified in 51% of tested isolates, and shared identical sequence to previously identified haplotype (H15). *ToxB* and its homolog *toxb* were present in 97% and 93% of tested isolates, respectively. *Ptr* in Tunisia lacked Ptr ToxC activity, and none of the tested isolates induced the specific symptoms of that effector. Race 7 and the atypical isolates dominated the Tunisian *Ptr* population, while races 2, 4, and 5 were found at low percentages. In conclusion, *ToxB* and its homolog were the most dominant genes in *Ptr* from Tunisia, and the majority of the isolates induced necrosis and chlorosis on Ptr ToxA and Ptr ToxB susceptible wheat genotypes. However, only about half of that necrosis can be attributed to *ToxA* presence, this result necessitates further research to investigate the prevalence of additional necrotic effector(s).

Terminology: in this paper, *Pyrenophora tritici–repentis* abbreviated as *Ptr*, the effectors are referred to by Ptr ToxA, Ptr ToxB and Ptr ToxC, and the genes coding for them are written in italic as *ToxA*, *ToxB*, and *ToxC*, respectively.

## Introduction

Tan spot of wheat is an important foliar disease found in major wheat-growing regions throughout the world. The causing pathogen is the ascomycete fungus *Pyrenophora tritici–repentis* (Died) (anamorph *Drechslera tritici–repentis*, Died) (*Ptr*), a necrotroph that infects both bread (*Triticum aestivum*) and durum wheat (*T. turgidum*). Tan spot is a disease of relatively recent history; it emerged as a threat to wheat over the last 40 years (Friesen et al., 2006). The wide adoption of minimum tillage practices and widespread cultivation of *Tsn1*-carrying wheat have likely caused a spike in its incidence and severity throughout the world (Lamari et al., 2005a). Beside its wheat host, *Ptr* was isolated from several grass species (Krupinsky, 1982; Ali and Francl, 2003), but it is known to cause severe damage only to wheat, although moderate symptoms were observed on other gramineous hosts (Morrall and Howard, 1975; Krupinsky, 1982; Summerell and Burgess, 1988). Lately, a specific interaction between *Ptr* and barley was evident, and confirmed the ability of *Ptr* effectors to interact specifically with additional hosts beside wheat (Aboukhaddour and Strelkov, 2016; See et al., 2019).

Tan spot is a stubble-born disease and the causing fungus overwinters as pseudothecia on wheat residue. Early in the growing season, infection by ascospores and conidia occur. When the spores land on susceptible wheat leaf under conducive conditions of continuous wetness and cool temperature, the spores germinate and penetrate through the epidermal cells or the stomata to invade the epidermal layer where the hypha grow intracellularly (inside cells). Then the fungus will invade the mesophyll layer intercellularly (between cells) causing damage to the organelle beyond its advancing hypha as results of necrotrophic effectors secretion by the pathogen (De Wolf et al., 1998; Aboukhaddour et al., 2011). Soon after the conidia germinate it secretes its necrotrophic effectors, previously known as host-selective toxins, that kill the plant cells so the fungus can acquire its nutrients from the dead cells. The infection manifests itself as tan colored necrotic or chlorotic lesions depending on the effector produced by the pathogen and the susceptibility gene in the host.

*Ptr* follows the inverse gene-for-gene interaction with its wheat host. In that, the unique interaction is genetically mediated between a dominant virulence from the pathogen side and a dominant susceptibility from the host side (Lamari and Strelkov, 2010). *Ptr* produces several necrotrophic effectors, three are identified as Ptr ToxA, Ptr ToxB, and Ptr ToxC. The Ptr ToxA protein is the only identified necrosis inducing effector in *Ptr*, and was the first proteinaceous necrotrophic effector reported in a fungal species (Ballance et al., 1996). It is encoded by a single copy gene, the *ToxA* gene (Ballance et al., 1996; Ciuffetti et al., 1997). On the other hand, Ptr ToxB and Ptr ToxC each induces chlorosis but on different wheat genotypes carrying different susceptibility genes to these two effectors. Ptr ToxB is the second proteinaceous effector identified in *Ptr*, and is encoded by a multi-copy gene, the *ToxB* gene (Strelkov et al., 1999; Martinez et al., 2001). Ptr ToxC exact nature and its coding gene (s) has not precisely identified, and an earlier research suggested that Ptr ToxC is a low-molecular-mass molecule (Effertz et al., 2002). Both *ToxA* and *ToxB* have homolog sequences in other related fungal species. *ToxA* identical sequence with 99.7% match was found in the wheat pathogen, *Stagonospora nodorum* (Friesen et al., 2006), and homolog of this gene was identified in the maize pathogen *Cochliobolus heterostrophus* (Lu et al., 2015) and in *Bipolaris sorokiniana*, which infect both wheat and barley (Stukenbrock and McDonald, 2007; McDonald et al., 2018). Yet in the same *Ptr* species, there are no homologs of this gene in isolates lacking the ability to produce the Ptr ToxA effector, and only the producing isolates carry the single copy *ToxA* gene (Aboukhaddour et al., 2009). On the other hand, homologs of *ToxB* were found in other fungal species of *Cochliobolus*, *Alteranaria*, and *Pyrenophora* (Andrie et al., 2008; Andrie and Ciuffetti, 2011), and in the *Ptr* isolates that do not produce the active Ptr ToxB protein.

Based on the pathogen ability to secrete combinations of these three effectors, eight different races have been identified worldwide and designated as race 1 through 8 (Lamari et al., 2003). Races 2, 3, and 5 produces one effector each Ptr ToxA, Ptr ToxC, and Ptr ToxB, respectively. Races 1, 6, and 7 produces combination of two effectors each, (Ptr ToxA + Ptr ToxC), (Ptr ToxB + Ptr ToxC), and (Ptr ToxA + Ptr ToxB), respectively. Race 8 is the most complex and produces the three effectors, while race 4 is the non-pathogenic and incapable of producing any of these effectors.

Research over the last 30 years in Canada, USA, South America, and Australia showed that Ptr ToxA-producing isolates of races 1 and 2 are the most predominant in these regions (Lamari et al., 1998; Ali and Francl, 2003; Friesen et al., 2005; Lamari et al., 2005b; Engle et al., 2006; Singh et al., 2007; Ali et al., 2010; Antoni et al., 2010; Leisova-Svobodova et al., 2010; Lepoint et al., 2010; Gamba et al., 2012; Aboukhaddour et al., 2013; Abdullah et al., 2017a; MacLean et al., 2017). On the other hand, Ptr ToxB-producing isolates have not been reported in Australia and New Zealand (Antoni et al., 2010; Weith, 2015), and were rarely found in the Americas (Aboukhaddour et al., 2013). Yet, *Ptr* race structure in regions encompassing the wheat center of origin, where wheat cultivations goes back thousands of years, like in the Middle East, North Africa, and the Caucasus regions, a high complexity in *Ptr* virulence is present, with all the eight races and effectors found and more likely to be identified (Lamari et al., 2005b; Aboukhaddour et al., 2009).

In North Africa, *Ptr* race structure was previously investigated in both Algeria and Morocco, and while Ptr ToxA-producing isolates dominated the Algerian fields as found by Benslimane et al. (2011), races 5 and 6 that lack the ability to produce Ptr ToxA, were the only detected races in Algeria in previous survey in 1993 (Lamari et al., 1995; Strelkov et al., 2002). In Morocco, races 5 and 6, Ptr ToxB-producers, composed the majority of *Ptr* isolates (Gamba et al., 2017). This study aims at investigating the *Ptr* race composition in Tunisia, an important wheat growing region in North Africa, to get more understanding of *Ptr* virulence in this area. The *ToxA* gene sequences from *Ptr* Tunisian isolates was analyzed in this study and compared to already published sequences from different parts of the world.

## Materials and Methods

### Fungal Isolates

**Figure 1:**
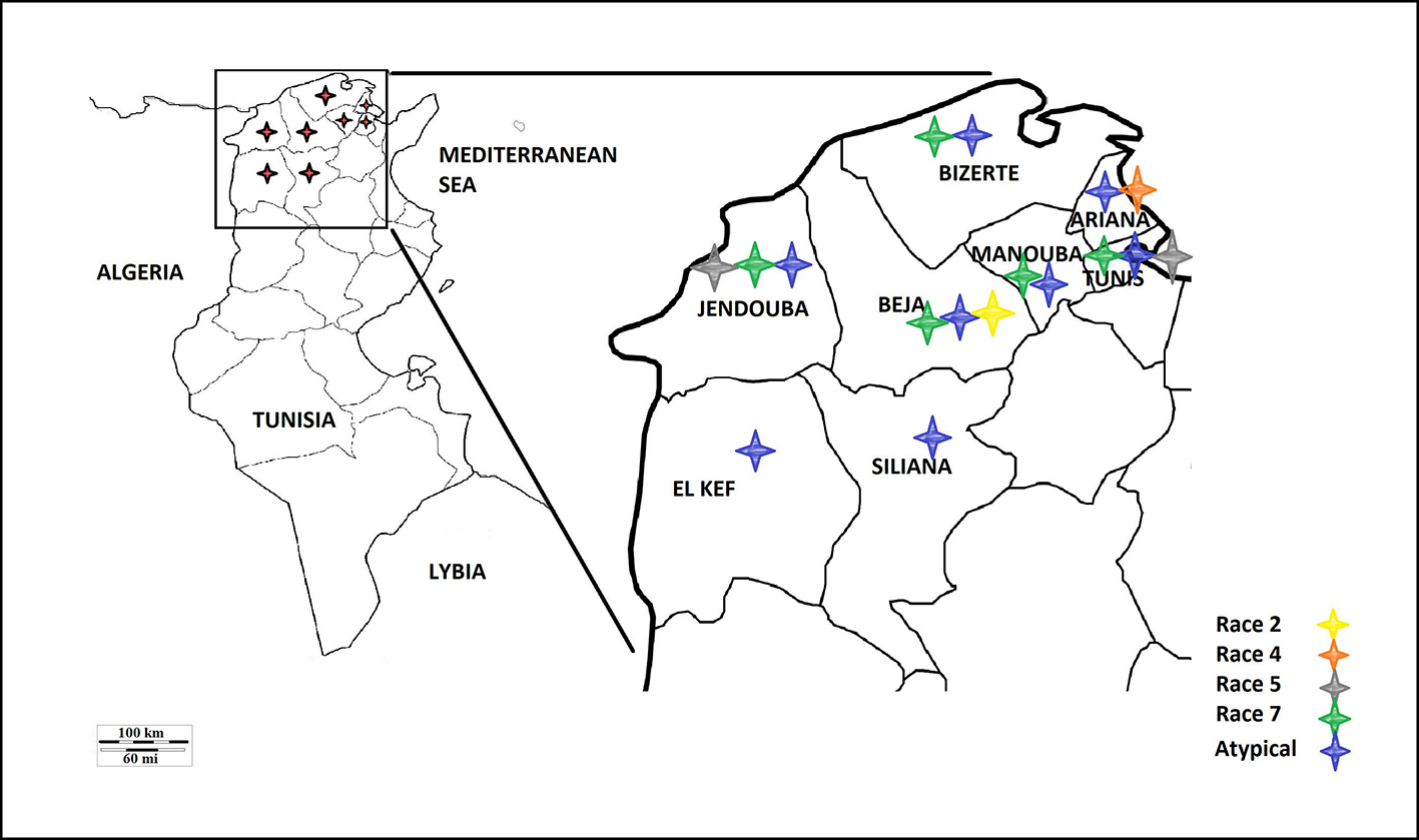
Map of Tunisia and the different *Ptr* races identified.

A survey of bread and durum wheat crops in Northern Tunisia, where wheat is growing, was conducted in 2017–2018 growing season to evaluate leaf spot incidence and severity. Leaf samples with typical tan spot symptoms were collected from individual wheat fields in the eight major wheat-growing areas. In total, 45 fields were surveyed in two different climatic regions, sub-humid (Bizerte, Beja) and semi-arid region of northern Tunisia (Tunis, Ariana, Manouba, Jendouba, Siliana and El Kef) (**Table 1**, **Figure 1**).

**Table 1:** Isolate code, geographic origin, host, PCR reaction, and race of *Ptr* isolates collected from different locations of Tunisia, and analyzed in this study.

Isolate^*^	Province	Host	PCR reaction			Race
			*ToxA*	*ToxB*	*toxb*	
T126-1	Ariana	DW	−	−	−	R4
T128-1	Ariana	DW	−	+	+	Atypical
T132-2	Beja	DW	+	−	−	R2
T133-3-4	Beja	DW	−	+	+	Atypical
T174-1	Beja	BW	−	+	+	Atypical
T177-1	Beja	BW	−	+	+	Atypical
T177-3	Beja	BW	−	+	+	Atypical
T177-4	Beja	BW	−	+	+	Atypical
T178-3	Beja	BW	−	+	+	Atypical
T172-2	Beja	DW	+	+	+	R7
T130-2	Beja	DW	+	+	+	R7
T130-4	Beja	DW	+	+	+	R7
T135-1	Beja	DW	+	+	+	R7
T173-4	Beja	DW	−	+	+	Atypical
T173-5	Beja	DW	+	+	+	R7
T173-9	Beja	DW	+	+	+	R7
T176-1	Beja	BW	+	+	+	R7
T176-2	Beja	BW	+	+	−	R7
T176-3	Beja	BW	+	+	+	R7
T178-1	Beja	BW	+	+	+	R7
T178-2	Beja	BW	+	+	+	R7
T179-1	Beja	BW	+	+	+	R7
T179-2	Beja	BW	+	+	+	R7
TB1-1	Beja	DW	+	+	+	R7
TB1-2	Beja	DW	+	+	+	R7
T165-1	Bizerte	DW	−	+	+	Atypical
T165-2	Bizerte	DW	−	+	+	Atypical
T167-0	Bizerte	DW	−	+	+	Atypical
T20-1	Bizerte	DW	−	+	+	Atypical
T20-2	Bizerte	DW	−	+	+	Atypical
T169	Bizerte	DW	+	+	+	R7
T17-2	Bizerte	DW	+	+	+	R7
T25-4	Bizerte	DW	+	+	+	R7
T25-7	Bizerte	DW	+	+	+	R7
T44-1	Bizerte	DW	+	+	+	R7
T44-4	Bizerte	DW	+	+	+	R7
T143-6	Jendouba	DW	−	+	+	Atypical
T143-7	Jendouba	DW	−	+	+	R5
T152-2	Jendouba	DW	−	+	+	Atypical
T61-19	Jendouba	DW	−	+	+	Atypical
T6-2-1	Jendouba	DW	−	+	+	Atypical
T64-39	Jendouba	DW	−	+	+	R5
T65-44	Jendouba	DW	−	+	+	Atypical
T70-66	Jendouba	DW	−	+	+	Atypical
TE6-6.2B	Jendouba	DW	−	+	+	Atypical
TE6-6.6B	Jendouba	DW	−	+	+	Atypical
TJ2-1	Jendouba	DW	−	+	+	Atypical
T146-5	Jendouba	DW	+	+	+	R7
T181-1	Jendouba	DW	+	+	+	R7
T2-1-6	Jendouba	DW	+	+	+	R7
T2-5-2	Jendouba	DW	+	+	+	R7
T75-1	Jendouba	DW	+	+	+	R7
TPTR3-1	Jendouba	DW	+	+	+	R7
T62-28-4	Jendouba	DW	+	+	+	R7
T157-2	El Kef	DW	−	+	−	Atypical
T157-3	El Kef	DW	−	+	+	Atypical
T157-5	El Kef	DW	−	+	+	Atypical
T102-1	Manouba	DW	−	+	+	Atypical
T39-3	Manouba	DW	−	+	+	Atypical
T39-5	Manouba	DW	−	+	+	Atypical
T103-1	Manouba	DW	+	+	+	R7
T103-2	Manouba	DW	+	+	+	R7
TPtr 47-3	Siliana	DW	−	+	+	Atypical
TPtr 47-4	Siliana	DW	−	+	+	Atypical
T171-1	Tunis	DW	−	+	+	R5
T129-4	Tunis	DW	−	+	+	Atypical
T129-5	Tunis	DW	−	+	+	Atypical
T168-1	Tunis	DW	+	+	+	R7
T168-2	Tunis	DW	+	+	+	R7
T168-3	Tunis	DW	+	+	+	R7
T168-4	Tunis	DW	+	+	+	R7
T168-6	Tunis	DW	+	+	+	R7
T168-7	Tunis	DW	+	+	−	R7

*Isolates designation: T for Tunis, the number after the T, indicates the number of field from which isolates was collected, and the number after the dash line is denoted for the particular isolates included in this study.

DW, durum wheat; BW, bread wheat.

+, amplification of the toxin gene; −, no amplification of the toxin gene.

Leaves with visible lesions were cut into 2 cm long segments. These segments were then surfaces-sterilized in 1% sodium hypochlorite solution for 2 min followed by water rinse twice for 30 s in sterile distilled water, and were placed in Petri plates containing moistened Whatman^®^ No. 1 filter paper (Whatman International Ltd., Maidstone, UK). The plates were incubated for 24 h at room temperature under fluorescent light, then transferred to the dark and incubated for additional 24 h at 15°C in the dark. The segments were then exanimated under stereoscope and single conidia were transferred each to individual 9-cm diameter Petri plates filled with V8-potato dextrose agar (V8-PDA) (Lamari and Bernier, 1989a). Single spore cultures were used to generate inoculum as described below.

### Inoculum Preparation and Bioassays

Inoculum preparation was done as previously described (Lamari and Bernier, 1989a; Lamari and Bernier, 1989b), the *Ptr* culture were grown in darkness at room temperature for a week, then mycelial plugs (0.5 cm in diameter) were excised from the growing cultures and transferred individually into a 9-cm Petri plates filled with V8-PDA and were incubated for 5 days in the dark at room temperature or until the colonies were 4 cm in diameter. Sterile distilled water was added to each colony and the mycelium were flattened with the bottom of flamed sterilized glass tube and excess water was decanted. The plates were then placed under fluorescent light for 18 h at room temperature, followed by 24 h incubation period at 15°C in the dark. Conidia were harvested after flooding the cultures with sterile distilled water and dislodging the spores gently with a wire loop. The inoculum concentration was adjusted to 3000 conidia ml^−1^ using a Fuchs Rosenthal Counting Chamber (Hausser Scientific, Blue Bell, PA). One drop of Tween 20 (polyoxyethylene sorbitan monolaurate) was added per 100 ml of conidia suspension.

### Wheat Differential and Symptoms Rating

A differential set composed of four hexaploid wheat genotypes (“Glenlea,” 6B662, 6B365, and “Salamouni”) were used in this study. These four genotypes can differentiate between the known eight races of the pathogen. Seeds of each genotypes were sown in 10-cm-diameter plastic pots filled with Sunshine potting Mix (W.R. Grace and Co., Fogelsville, PA) at a rate of eight seeds by pot. One genotype was seeded per pot, and all treatments were replicated twice, and the whole bioassay was repeated one additional time. Pots were placed in a growth chamber at 16 h photoperiod (22°C day/18°C night) at 180 mmol m^−2^ s^−1^, for seed germination and seedling development. After 14 days, and when plants reached the two leaf stage, seedlings were inoculated and rated for symptoms development 6 days after inoculation. Wheat seedlings were sprayed with the conidial suspension [3000 conidia ml^−1^ and one drop of Tween 20 (polyoxyethylene sorbitan monolaurate) 100 ml^−1^] until runoff using a sprayer connected to an airline.

Immediately following inoculation, the seedlings were covered with plastic bag to maintain humidity. The seedlings were then transferred to a growth cabinet and kept at 20°C/18°C (day/night) with a 16 h photoperiod (180 mmol m^−2^ s^−1^). The three isolates Asc1 (race 1), D308 (race 3), and Alg3–24 (race 5), Ptr ToxA, Ptr ToxC, and Ptr ToxB-producers, respectively, were used in each inoculation as positive control for the symptoms corresponding to each effector.

### Genomic DNA Extraction

Genomic DNA (gDNA) was extracted using 40 mg of lyophilized mycelium. In order to produce mycelia, 3 ml of spore suspension ∼3000 conidia ml^−1^ were transferred to 250 ml Erlenmeyer flasks containing 100 ml of Fries liquid medium [5 g (NH)_4_C4H_4_O_6_, 1 g NH_4_NO_3_, 0.5 g MgSO_4_.7H_2_O, 0.13 g KH_2_PO_4_, 0.26 g K_2_HPO_4_, 30 g sucrose, 1 g yeast extract, 2 ml trace element stock solution] (Dhingra and Sinclair, 1986). Flasks were incubated in darkness at 20°C for 3 weeks. The mycelial mats were separated from the liquid medium by vacuum filtration through Whatman^®^ No. 1 filter paper. Mycelia mats were then flash-frozen in dry ice and lyophilized in a freeze-drier prior to DNA extraction.

The gDNA was extracted from each isolate using the Wizard^®^ Genomic DNA Extraction Kit (Promega Corporation, Madison, WI), according to the manufacturer’s protocol for plant material, with added steps one with phenol chloroform (1:1 v/v) followed by one extraction with chloroform. The DNA quantity and quality was measured using a NanoDrop ND-1000 spectrophotometer (Thermo Scientific, Wilmington, DE), and adjusted to a final concentration of 50 ng µl^−1^ in Tris-EDTA buffer.

### PCR Amplification of Effector Genes

Singleplex PCR analysis to detect the presence or absence of the effectors genes *ToxA* and *ToxB* and its homolog *toxb* in *Ptr* population in Tunisia was performed using gDNA from the 73 *Ptr* isolates tested in this study. Two *ToxA-*specific primer pairs were used to detect the presence or absence of *ToxA* gene (**Table 2**), one pair targeting the *ToxA*-ORF (ToxA1/ToxA2) and a second pair targeting a larger fragment contains *ToxA*-ORF and non-coding regulatory sequences (ToxA192/ToxA1155). *ToxB* primers (ToxB1/ToxB2) designed to amplify the ORF of this gene. The primers used for *toxb* amplification (toxb90-2F1/toxb90-2R1) were designed with Primer 3 software (Rozen and Skaletsky, 2000) based on sequences of *toxb* in a Canadian race 4 (GenBank accession No. AF483832.1) (**Table 2**). To confirm the results obtained by the singleplex PCR, a multiplex PCR was followed on representative Tunisian *Ptr* isolates of races 2, 4, 5, 7 and the atypical isolates. In addition, previously identified and confirmed isolates of races 1, 2, 3, 4, and 5 were included. In the multiplex PCR, primers specific to the chitin synthase 1 (*CHS1*) gene, a conserved gene in fungi, were added as a control test for the presence of fungal DNA. In addition, primers specific to *ToxA*, *ToxB*, and *toxb* in the multiplex were all adopted from Andrie et al. (2007) (**Table 2**).

**Table 2:** Primers used for amplification of the *ToxA*, *ToxB* and *toxb* genes in *Ptr* isolates.

Gene	Primer for singleplex PCR	Sequence	Estimated band size (bp)
*ToxA*	TOXA192	5′-CGT CCG GCT ACC TAG CAA TA-3′	964
	TOXA1155	5′-TTG TGC TCC TCC TTC TCG AT-3′	
*ToxA*	ToxA1	5′-GTC ATG CGT TCT ATC CTC G-3′	
	ToxA2	5′-CCT ATA GCA CCA GGT CGT CC-3′	294
*ToxB*	ToxB1	5′-GAC TAC CAT GCT ACT TGC TGT G-3′	
	ToxB2	5′-AAC AAC GTC CTC CAC TTT GC-3′	245
*toxb*	90-2F1	5′-AAG TGG TCA TTG CGA CTG G-3′	
	90-2R1	5′-CCT CCA CTT GCC AAA CTC TC-3′	157
Gene	Primer for multiplex PCR	Sequence	Estimated band size (bp)
*CHS-1*	CHS-79F	5′-TGGGGCAAGGATGCTTGGAAGAAG-3′	275
	CHS-354R	5′-TGGAAGAACCATCTGTGAGAGTTG-3′	
*ToxA*	TA51F	5′-GCGTTCTATCCTCGTACTTC-3′	573
	TA52R	5′-GCATTCTCCAATTTTCACG-3	
*ToxB*	TB71F	5′-GCTACTTGCTGTGGCTATC-3	232
	TB60R	5′-ACTAACAACGTCCTCCACTTTG-3′	
*toxb*	TB71F	5′-GCTACTTGCTGTGGCTATC-3	232
	TB58R	5′-TATGAATGATTGACTGGGGTTA-3′	

Singleplex PCR reactions were performed in a final volume of 50 µl using the Taq PCR core kit (Qiagen Inc. Ontario, Canada) with the following reagent concentrations: CoralLoad PCR buffer (1×); dNTP mixture (200 µM each); forward and reverse primers (0.2 µM each); Taq DNA polymerase (1.25 U/50 µl); 50 ng of gDNA template and the total volume of the PCR reaction adjusted to 25 µl with nuclease-free H_2_O. The amplification conditions consisted of initial denaturation step at 95°C for 2 min, followed by 30 cycles of 94°C for 50 s, 55°C for 50 s, and 70°C for 50 s with a final extension at 70°C for 7 min. All PCR amplicons (*ToxA*, *ToxB*, and *toxb*) were analyzed by gel electrophoresis through 1.5% agarose gels in 1× TBE buffer (89 mM Tris-borate, 10 mM EDTA, pH 8.0). The sizes of the PCR amplicons were estimated against a 1 kb plus ladder (Thermo Fisher Scientific, Canada) and visualized under UV light after staining with RedSafe dye (Intron Biotechnology Inc. Seoul, Korea). PCR amplicons were purified and sequenced by Macrogen (Rockville, MD, USA). *Ptr* isolates Asc1 (race 1), 90-2 (race 4), and Alg3–24 (race 5) were used as positive controls for *ToxA*, *toxb*, and *ToxB* genes, respectively.

For multiplex PCR, the conditions were adapted from Andrie et al. (2007) with slight modifications. PCR reactions were performed in a final volume of 50 µl using the DreamTaq Green PCR Master Mix (Thermo Fisher Scientific, Canada) with the following reagent concentrations: 1 × Green Taq buffer; 200 µM each dNTP; 0.2 µM each primer except for TA51F and TA52R primers, µM 0.4 from each primer were used; 1.5 U Dream Taq DNA polymerase; ∼20 ng of gDNA template and the total volume of the PCR reaction adjusted to 50 µl with nuclease-free H_2_O. PCR amplification included an initial denaturation step at 94°C for 3 min, followed by 35 cycles: 94°C for 45 s, 58°C for 30 s, 72°C for 1 min, and a final extension at 72°C for 10 min.

### Sequence Analysis for *ToxA*, *ToxB*, and *toxb*

*ToxA*, *ToxB*, and *toxb* amplifications were done using primers combination ToxA192/ToxA1155, ToxB1/ToxB2, and 90-2F/90-2R, respectively (**Table 2**). PCR amplicons were sequenced in both directions with the forward and reverse primers and the generated sequences were compiled and assembled manually into contigs using the GeneDoc program v2.5.010 (Nicholas et al., 1997). The initial nucleotide sequence alignments were done with the Clustal-X program (Thompson et al., 1997) and the resulting alignments were refined by eye with the GeneDoc program.

The consensus *ToxA* sequence was used to interrogate the online resource Basic Local Alignment Search Tool (BLAST: http://www.ncbi.nlm.nih.gov/BLAST/; Altschul et al., 1990) to retrieve similar sequences from NCBI database to have significant representatives from each *ToxA* haplotype investigated in the current study. **Supplementary Table 1** shows GenBank accession numbers of *ToxA*, *ToxB*, and *toxb* sequences from Tunisian *Ptr* isolates (MN052875 to MN052896), Canadian *Ptr* isolates (MN062685 to MN062700), and other sequences retrieved from GenBank for the analysis (EF108451 to EF108463, MH511822, MH511823, and KX816409).

## Results

### Race Characterization

**Figure 2:**
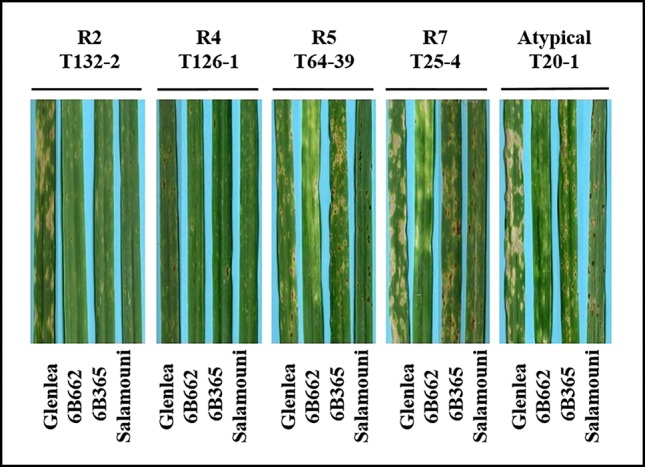
Reaction of differential genotypes to inoculation with representative *Ptr* isolates collected from Tunisia.

A total of 73 single spore *Ptr* isolates (61 from durum wheat and 12 from common wheat) were recovered and characterized in this study (**Table 1**). These isolates were tested for ability to induce symptoms on the differential wheat lines in this study. The majority of the tested isolates 93% (68) were able to induce necrosis on the wheat differential line “Glenlea” and Chlorosis on 6B662, but 6B365 and “Salamouni” exhibited a resistant reaction (**Figure 2**), a symptom typical for race 7.

Three isolates (4%) were designated as race 5, since they could induce chlorosis on 6B662, but were avirulent on the rest of tested genotypes (**Figure 2**). Only one isolate (1%) was classified as race 2, and was able to cause necrosis on “Glenlea” but remain avirulent on the rest of tested genotypes (**Figure 2**). A single isolate (1%) was classified as race 4, a non-pathogenic, and was avirulent on all tested wheat genotypes (**Figure 2**). “Salamouni” and 6B365 exhibited resistant reaction to all tested Tunisian isolates (**Figure 2**). “Salamouni” is known as a resistance genotype for *Ptr*, and 6B365 is susceptible to Ptr ToxC-producing isolates. In each bioassay, the three isolates ASC1, D308, and Alg3-24 representing races 2, 3, and 5, respectively showed typical symptoms for the race each represents on the differential set.

### Presence of *ToxA*, *ToxB*, and *toxb* Genes in *Ptr* Population in Tunisia

**Figure 3:**
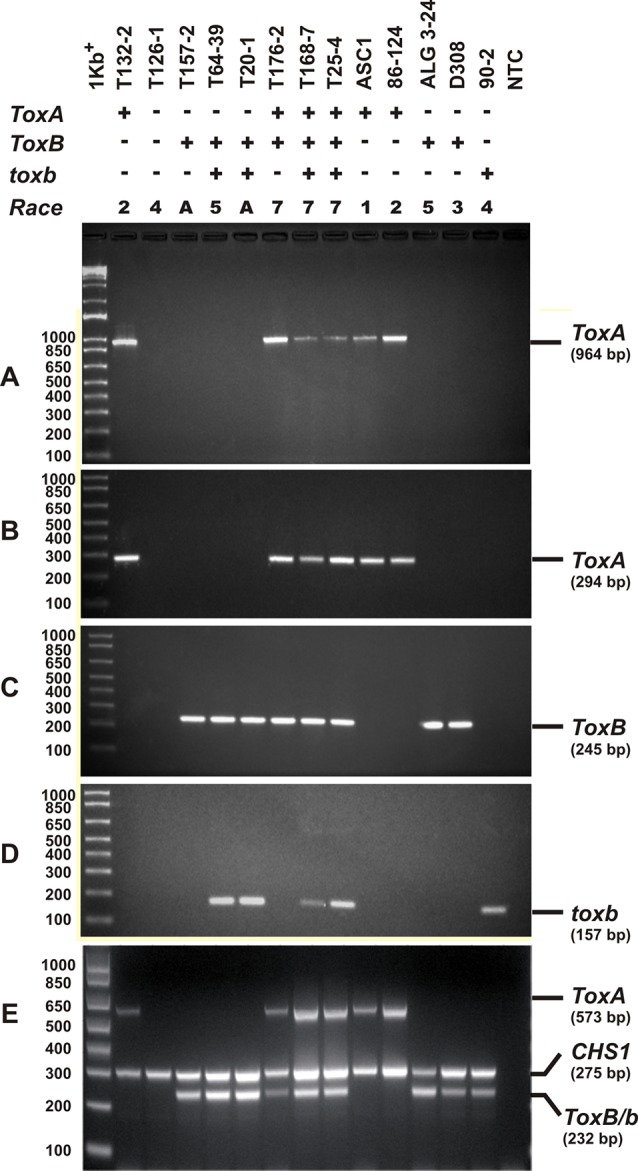
PCR amplification assays with primer sets specific for *ToxA*, *ToxB*, and *toxb* genes. A subset of *Ptr* isolates used as control, these are ASC1, 86-124, ALG3-24, D308, and 90-2, and they represent races 1, 2, 5, 3, and 4, respectively. Genomic DNA was subjected to PCR analysis with *ToxA*-specific primers [ToxA192/ToxA1155 **(A)**, ToxA1/ToxA2 **(B)**], *ToxB*-specific primers (ToxB1/ToxB2) **(C)**, and *toxb*-specific primers (90-2F1/90-2R1) **(D)**. A multiplex PCR with specific primers to *ToxA*, *ToxB*, *toxb*, and chitin synthase 1 gene (*CHS1*) as an internal control for the presence of fungal DNA **(E)**. All PCR products were resolved in 1.5% agarose supplemented with RedSafe nucleic acids staining solution.

Singleplex PCR analysis to determine the presence or absence of two effectors genes *ToxA* and *ToxB*, and the homolog *toxb* was done on all the isolates tested above and repeated three independent times. An amplicon corresponding to *ToxA* gene was present in 51% (37) of the isolates (**Figures 3A, B**), and these isolates induced the tan necrosis on “Glenlea.” However, this gene was absent in 44% (32) of the tested isolates (**Figures 3A, B**), although these 32 isolates caused the typical necrotic symptoms on “Glenlea.” This represents an atypical result where the necrosis development on “Glenlea” cannot be attributed to Ptr ToxA presence, and these isolates therefore are designated here as atypical (**Table 1**). An amplicon corresponding to *ToxB* was found in 97% (71) of tested isolates, but was not detected in races 2 and 4 isolates (**Figure 3C**). As expected, all the isolates that amplified *ToxB*, induced chlorosis on 6B662. Its homolog the *toxb* gene, was also amplified in 93% (68) of tested isolates and was not detected in isolates of races 2 and 4 (**Figure 3D**). The multiplex PCR amplification patterns for isolates from races 2, 4, 5, 7, and the atypical were as expected (**Figure 3E**). *CHS-1* gene amplified from all tested isolates (**Figure 3E**), but *ToxA* amplicon was detected in isolates of races 2 and 7, and was absent in isolates from races 5, 4, and the atypical. The *ToxB* gene amplified from isolates of races 5, 7, and the atypical, but was absent in isolates of races 2, and 4 as expected (**Figure 3E**).

Based on the combined bioassay and PCR results, race 7 is the most predominant in Tunisia, with 36 (49%) isolates collected from wheat fields in five Tunisian provinces (Tunis, Manouba, Bizerte, Beja, Jendouda) (**Table 1**). These isolates caused the development of necrosis on the host differential “Glenlea” and chlorosis on 6B662, but were avirulent on 6B365 and “Salamouni” (**Figure 2**), and amplified both *ToxA* and *ToxB* genes (**Figures 3A–C, E**). The atypical isolates were as dominant, 44% (32) of the tested isolates, and induced the same symptoms on wheat differential as to race 7, but failed to amplify the *ToxA* gene (**Figures 3A, B, E**). These atypical isolates were collected from all surveyed provinces in Tunisia (**Figure 1**).

Three isolates (4%) collected from Tunis and Jendouba were designated as race 5, since they could induce chlorosis on 6B662, and were avirulent on the rest of the tested genotypes (**Figure 2**), and amplified *ToxB* but not *ToxA* (**Figures 3A–C, E**). A single isolate (1%) originated from Beja was classified as race 2, and was able to cause necrosis on “Glenlea” but remain avirulent on the rest of tested genotypes (**Figure 2**), and amplified *ToxA* only (**Figures 3A, B, E**). A single isolate (1%) originated from Ariana was classified as race 4, non-pathogenic, and failed to amplify *ToxA* or *ToxB* (**Figures 3A–C**).

### Sequence Analysis

#### ***ToxA*** Haplotypes

**Figure 4:**
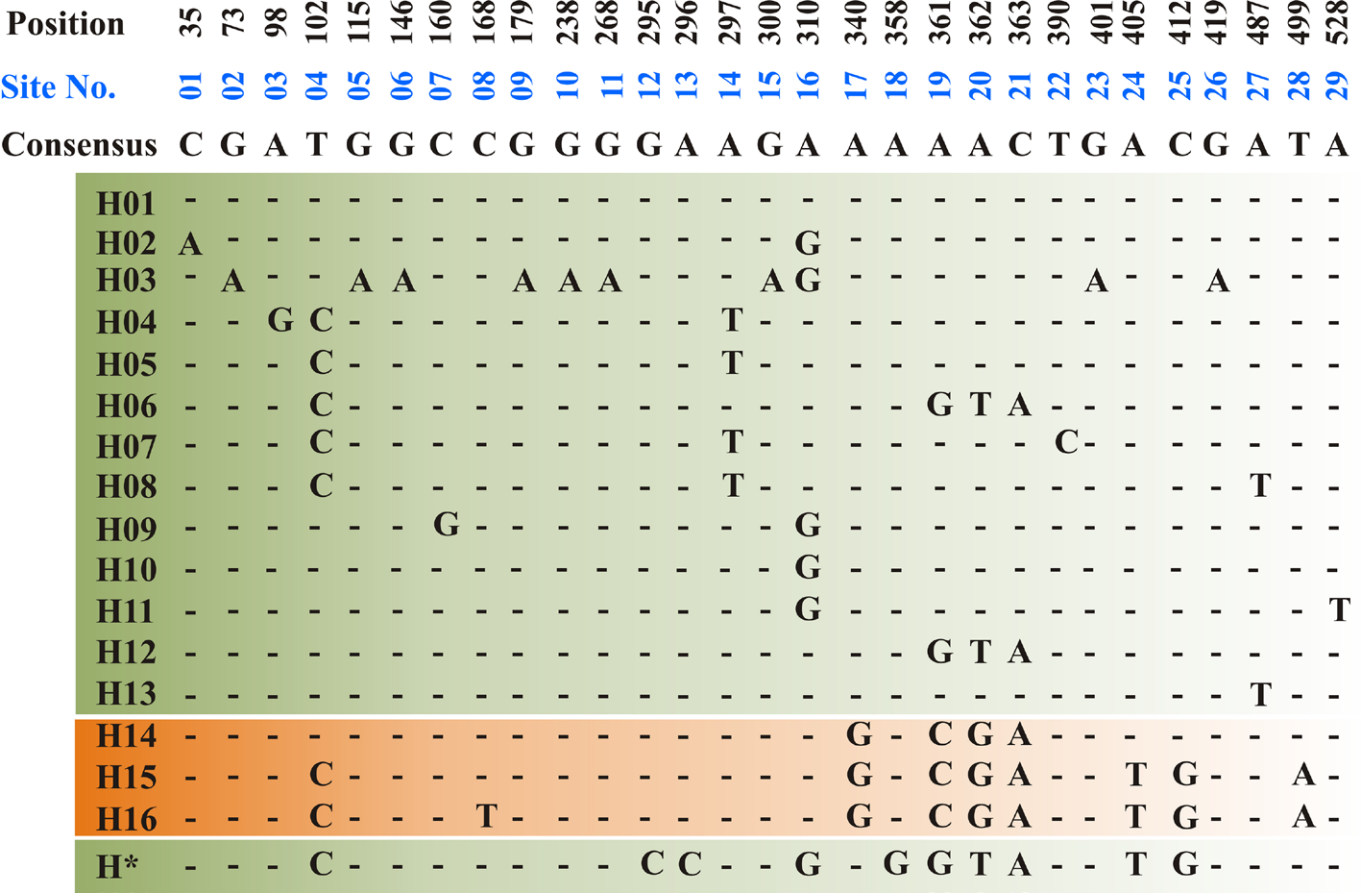
Haplotype alignment for *ToxA* gene in *S. nodorum* (H01–H13 — green background) and *Ptr* (H14–H16—orange background) as cited by Stukenbrock and McDonald, (2007). A potential new *S. nodorum* haplotype (H*) was reported in this study and originated from Australia. A total of 29 polymorphic site (01–29) was recorded and nucleotide positions are numbered relative to *ToxA* start codon. All Tunisian *Ptr* isolates investigated during the current study represent the H15 haplotype.

The *ToxA*-coding region includes two exons consisting of 534 bp separated by one intron of 50 bp. All sequenced amplicons in the current study showed identical sequence in both exon and intron regions. Sequence analysis of the Tunisian *ToxA* and previously published *ToxA* sequences from *Ptr* and *S. nodurm* (Stukenbrock and McDonald, 2007) showed 29 polymorphic sites distributed among 17 different haplotypes (14 haplotype in *S. nodorum* and 3 haplotypes in *Ptr*). Three different haplotypes (H14, H15, and H16) were found in *ToxA* sequences, and the same haplotype (H15) was the only haplotype found in the Tunisian isolates in this current study, and it was also the only *ToxA* haplotype found in Canadian *Ptr* isolates (MN062685 to MN062700) collected in 2010 from Alberta. In this study, *ToxA* sequence analysis reveals a novel haplotype from a *S. nodorum* isolate Fr15-02 that was deposited in GenBank by Australian researchers (GenBank accession MH511823), this potential new *in silico* identified haplotype (H*) is included in **Figure 4**.

#### ***ToxB*** and Its Homolog ***toxb***

*ToxB* gene and its homolog *toxb* from five isolates were sequenced (**Table 1**). The sequencing results showed that *ToxB* sequences in these five isolates were identical to *ToxB* gene in race 5 (Strelkov et al., 2006). In this study, *ToxB* and *toxb* sequences were found to be different at three positions at the DNA level similar to what previously reported as race 3 *toxb* (accession number AF483833) by Strelkov et al. (2006), and these three mutations in *toxb* gene translated into change in two residues in *Ptr* ToxB protein at positions 58 and 59. Valine (V58) and glycine (Q59) in the functional *Ptr* ToxB protein were replaced by Leucine (L58) and Alanine (A59) in the non-functional homolog encoded by *toxb*.

## Discussion

**Figure 5:**
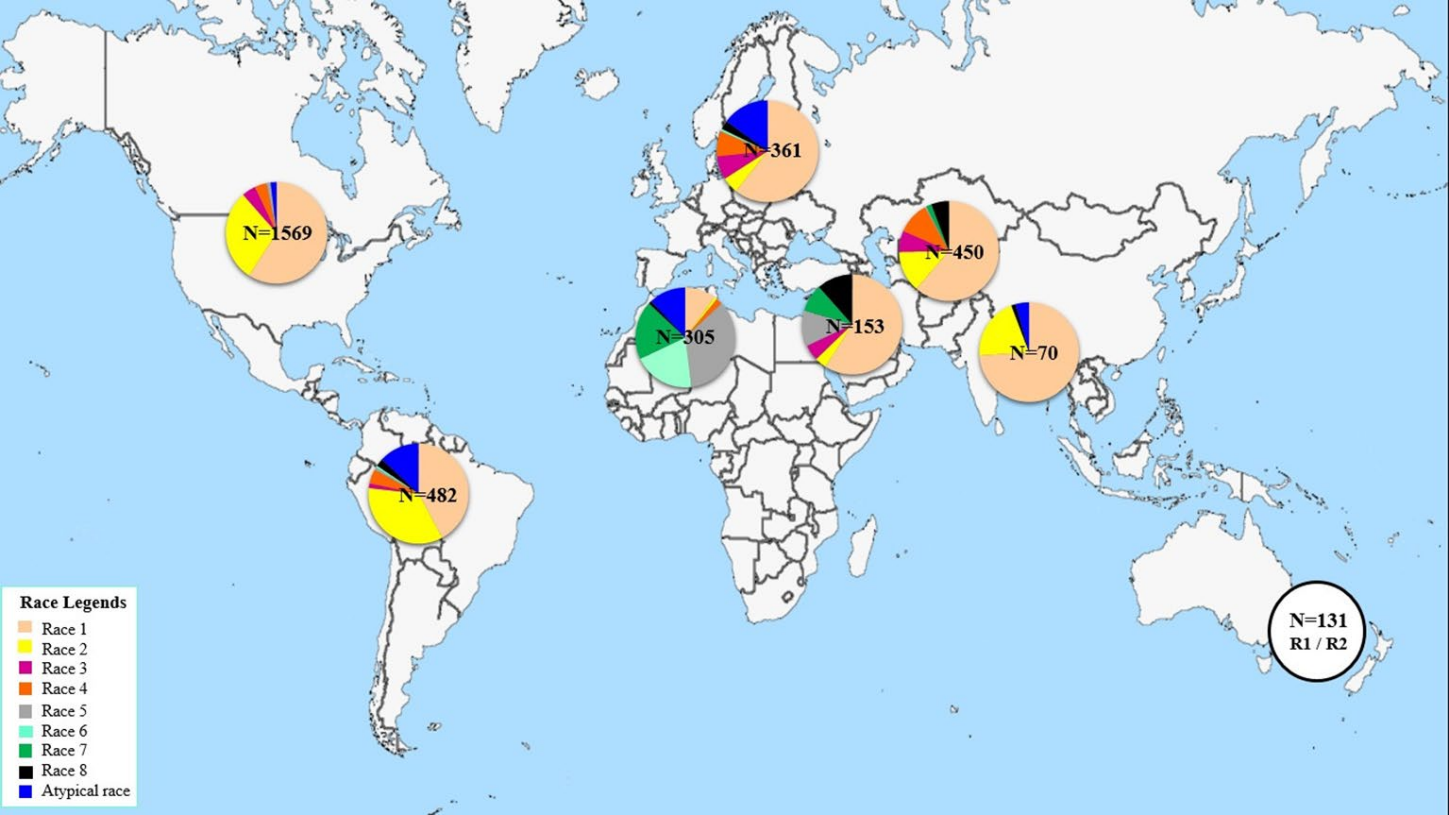
Distribution of *Ptr* races across worldwide geographical regions since 1989.

This study aims at determining the race structure of *Ptr* population in Tunisia, the prevalence of its effector genes, and its *ToxA* haplotypes. To our knowledge this is the first report of *Ptr* in Tunisia and *ToxA* haplotypes in North Africa. Here, a summary of *Ptr* race structure around the world since 1989 utilizing the race system described by (Lamari et al., 2003) is presented in **Figure 5** and **Supplementary Table 2**. In brief, race 1 of the pathogen is the most predominant race around the world. It is prevalent in the Americas, Europe, North and South Asia, and Russia. However, *Ptr* population in North Africa and in some regions in the caucuses is unique, with Ptr ToxB-producing isolates (races 5, 6, 7, and 8) dominating these regions, but are absent or rarely found in the rest of the world (**Figure 5**). Race 2, ranks second in frequency after race 1 in the Americas, and in Asia, but it was found at low frequencies in Europe, the Caucuses, and in North Africa (Canada and USA) (**Figure 5**). In Australia and New Zealand, the exact identity of races 1 or 2 remains to be investigated but a combination of these two races is possible, and this concluded based on the presence of *ToxA* but not *ToxB* in the pathogen populations in these regions (Antoni et al., 2010; Weith, 2015). Races 3 and 4, do occur in various geography but at low frequencies, except when the fungus is recovered from grasses, then race 4 is the most prevalent one (Ali and Francl., 2003). There are several reports on isolates that do not fit within the known 8 races, and were denoted here as atypical (**Figure 5**).

North America so far is the most investigated region in the world with over 1500 isolates being characterized mainly as races 1 or 2 over the last 30 years (**Figure 5**). Yet, up to 5 different races were recovered from a single visit to a single field in the wheat center of origin region (Lamari et al., 2005b). Six races were identified from a small collection of only 55 isolates in Algeria (Benslimane et al., 2011). Information on *Ptr* race structure in western Europe is limited, likely the pathogen is not as important in that region, but few reports from eastern, eastern south, and central Europe, showed dominance of race 1 isolates, and few isolates amplified the *ToxB* gene (Sarova et al., 2005; Leisova-Svobodova et al., 2010; Abdullah et al., 2017b). Russian *Ptr* populations from Northwest and North Caucasus territory harbor all the eight races (Mironenko et al., 2019).

In this current study, four different races (races 2, 4, 5, and 7) were detected beside one atypical virulence group that did not fit under the current race system (**Table 1**). These atypical isolates induced the same symptoms as race 7 isolates, but lacked the *ToxA* gene (**Table 1**). Both race 7 and the atypical isolates represented 93% of the pathogen population in Tunisia, with race 7 at 49% and the atypical isolates at 44%. Races 2, 4, and 5 occurred at a very low frequencies, 1%, 1%, and 4%, respectively. In terms of effectors prevalence, *ToxB* and its homolog, *toxb*, predominated the Tunisian *Ptr* population, and occurred at 97% and 93%, respectively, while *ToxA* occurred in 51% of the tested isolates.

*Ptr* race composition in Tunisia generally was similar to the races present in regions in close proximity to the wheat center of origin, where both Ptr ToxA and Ptr ToxB-producing isolates are reported (Lamari et al., 2005b). However, unlike previous results from North Africa (Benslimane et al., 2011; Gamba et al., 2017), *Ptr* in Tunisia in this study lacked races 1, 6, and 8. Yet, race 3 was never reported in previous studies in North Africa (Lamari et al., 1995; Benslimane et al., 2011; Gamba et al., 2017) or in this current study.

In Morocco, a more recent study investigated a larger collection of 135 single spore isolates, and showed the presence of races 1, 5, 6, and 7 (Gamba et al., 2017). While races 1 and 7 dominated Algerian wheat, these races were reported at low percentage in Morocco, as races 5 and 6 were the most predominant ones, and together accounted for 91% of detected isolates. In this study, race 7 occurred at 50% which is very close to its frequency in Algeria at 40%. Similar to what was reported in Morocco, our results, showed predominance of Ptr ToxB-producing isolates at 97%, although the race designation varied between the two countries. Previous survey conducted in eastern Algeria in 1993, showed that *Ptr* isolates were grouped in either race 5 or race 6 (Lamari et al., 1995; Strelkov et al., 2002). Only in 2011, races 1 and 4 were reported from Algeria (Benslimane et al., 2011), but there is no additional survey between these two studies in Algeria to explain the differences in these results.

Although Morocco, Algeria, and Tunisia are all in North Africa and generally share similar geography and common landrace, but finding conflicting results on the diversity or composition of *Ptr* races in close by countries is not uncommon. For example, in Azerbaijan, where combinations of all effectors and most of the known races were detected (Lamari et al., 2005b), only one or two races (races 1 and 2) were found in the neighboring regions such as Uzbekistan, Kazakhstan, Kryghyztan, and Iran (Lamari et al., 2005b; Momeni et al., 2014). The conflict in results regarding race composition among these neighboring regions was in part attributed to the host from which these isolates were recovered. In Azerbaijan, local varieties and land races are often planted, where in Uzbekistan, Kazakhstan, Kryghyztan, and Iran, modern cultivars developed by the International Maize and Wheat Improvement Center (CIMMYT) and the International Center for Agricultural Research in the Dry Areas (ICARDA), are planted often rather than local varieties (Lamari et al., 2005b; Momeni et al., 2014). Genetic diversity in *Ptr* from a global collection, showed a significant differentiation between Ptr ToxA-producing and non-producing isolates, and suggested that host-specificity imposed by necrotrophic effectors may lead to the differentiation among races (Aboukhaddour et al., 2011), as majority of Ptr ToxA-non producers were collected from durum (Aboukhaddour et al., 2011). Indeed, the three race 5 isolates identified in this current study were collected from durum, and in the previous studies in Algeria and Morocco, all races 5 and 6 were recovered from durum only (Lamari et al., 1995; Gamba et al., 2017).

The variation among races in North Africa is hard to attribute only to geography, yet the number of fields sampled, recovered isolates, and years of survey is relatively small to draw a final conclusion. One common finding in North Africa, is the lack of race 3, a Ptr ToxC-producer, in this region, and this race however was the most frequent in Syria (Lamari et al., 2005b). In Tunisia, there was a total absence of Ptr ToxC activity that remain to be explained, yet races 6 and 8 were reported in North Africa (Lamari et al., 1995; Benslimane et al., 2011; Gamba et al., 2017) and these races produces the Ptr ToxC effector.

In this study, we report on the finding of atypical isolates occurring at a high percentage of 44% in *Ptr* population in Tunisia, these isolates have the ability to cause necrosis on “Glenlea” a *Tsn1*-carrying genotype, but lack the *ToxA* gene. Therefore, could not fit under the current race system describe by Lamari et al. (2003). Recently, a similar finding describing predominant necrosis on “Glenlea” by *Ptr* isolates that lack *ToxA* gene was reported in northern and western Siberian pathogen population (Mironenko et al., 2019). This indicates the widespread of additional races and necrotic factors in *Ptr* populations.

Ptr ToxA, so far is the only described necrotic causing effector, its gene, *ToxA*, has a very conserved sequence and believed to be horizontally transferred into *Ptr* genome from a related species *S. nodorum* (Friesen et al., 2006). *ToxA* sequence is highly conserved in *Ptr*, and only one haplotype (H14) was detected when a collection of 54 *Ptr* isolates from geographically diverse pathogen populations were screened (Friesen et al., 2006). Stukenbrock and McDonald (2007) reported three different haplotypes in a collection of 61 *Ptr* isolates collected from different geographic regions including the 54 *Ptr* isolates previously investigated by Friesen et al. (2006). These three *ToxA* haplotypes denoted H14 (59), H15 (1), and H16 (1), where number in parentheses represent the number of isolates belonging to each haplotype. In another study by Aboukhaddour et al. (2013), 16 *Ptr* isolates collected in 2010 from Alberta, Canada were all found to have *ToxA* H15 (MN062685 to MN062700). In contrast to *ToxA* haplotypes in *S. nodorum* which has several haplotypes, only 3 *ToxA* haplotypes were recorded in *Ptr* (Stukenbrock and McDonald, 2007; McDonald et al., 2013). In this study, *ToxA* sequence comparative analysis showed that, all Tunisian *Ptr* isolates were found to have *ToxA* sequences identical to the previously identified *ToxA* haplotype 15 (H15: **Figure 4**). *ToxA* haplotyping is well studied in *S. nodorum* (Friesen et al., 2006; Stukenbrock and McDonald, 2007; McDonald et al., 2013), but poorly studied in *Ptr* and to our knowledge, this is the first *Ptr ToxA* genotyping analysis in Tunisia and North Africa. The *ToxA* haplotype in Tunisian isolates (H15) is identical to the USA isolate Pt-1C (Ciuffetti et al., 1997), and the Canadian *Ptr* isolates (MN062685 to MN062700).

Before this current study, 20 different *ToxA* haplotypes were identified, 3 in *Ptr* and 17 in *S. nodorum* (Stukenbrock and McDonald, 2007; McDonald et al., 2013). However, additional homolog sequence of *ToxA* in *S. nodorum* isolate Fr15-02 was found in the GenBank (Accession MH511823) and was submitted by The School of Molecular and Life Sciences in Western Australia, Australia. In this study, we found that this submitted sequence (Accession MH511823) does not match any of the previously identified haplotypes (**Figure 4**). This indicates additional haplotypes of ToxA gene. The high *ToxA* sequence diversity in *S. nodorum* (14 haplotypes) in comparison to the low sequence diversity in *Ptr* (3 haplotypes) confirmed previous conclusions on a more recent acquisition of *ToxA* by *Ptr* genome (Friesen et al., 2006; Stukenbrock and McDonald, 2007).

Unlike the conserved sequence of *ToxA* in Tunisian *Ptr*, *ToxB*, and its homolog *toxb* were both amplified in the same isolate from 93% of tested isolates, and was not detected in races 2 and 4 isolates. The Tunisian race 4 in this study did not amplify *toxb*, however, the primers for *toxb* used in this study were designed based on the sequence of *toxb* homolog in a Canadian race 4 isolate, and variation among different *toxb* homologs from different regions or races may render the PCR negative. Usually, *toxb* which encodes non-functional protein is found in isolates of races 3 and 4, that lack the active *ToxB* gene (Strelkov et al., 2006), but here to find the *ToxB* and its homolog *toxb* in the same Tunisian isolates of races 5, 7, and the atypical isolates is an interesting finding reflecting on a more complex evolutionary history of *ToxB* and its homolog in this species.

No doubt, that the *Ptr* race system greatly impacted our understanding of necrotrophic effectors–host interaction and gave us the ability to differentiate among the eight races of *Ptr*. However, the need to expand it and identify additional effectors was discussed already by the scientists who established this system in the first place (Lamari et al., 2003). Obviously, the ability to identify the full races of a pathogen is limited by the differential set in use, and additional races, and effectors most likely exist in *Ptr*. In this study, the ability to cause necrosis without the presence of *ToxA* gene, can be explained by the presence of additional pathogenicity gene(s) that await further characterization.

## Data Availability Statement

All datasets generated for this study are included in the article/**Supplementary Material**.

## Author Contributions

SK performed most of the work in this manuscript and drafted the manuscript, MH performed sequence analysis and helped in aspects of molecular work, TD provided considerable help in purifying and growing the cultures and assisted in all experimental aspects. MC and RA conceived the experiment and RA with MH revised and edited the work. All authored reviewed and contributed to the manuscript. Survey and single spore isolation was done in Tunisia and rest of the work was performed in Lethbridge Research Centre in Agriculture and Agri-Food Canada.

## Funding

Funding from the University of Carthage to SK, and funding from Agriculture and Agri-Food Canada and Alberta Wheat Commission and Saskatchewan Wheat Development Commission to RA. The funding bodies were not involved in the design of the experiments and collection, analysis, and interpretation of data, or in the writing of this manuscript.

## Conflict of Interest

The authors declare that the research was conducted in the absence of any commercial or financial relationships that could be construed as a potential conflict of interest.
