# Supplementary material for: Pyrenophora tritici–repentis in Tunisia: Race Structure and Effector Genes
Source: Front Plant Sci. 2019 Dec 18;10:1562. doi: 10.3389/fpls.2019.01562 (PMC6930679; doi:10.3389/fpls.2019.01562)
Supplement: Supplementary file 1 [file Table_1.pdf]

Supplementary table 1: *ToxA* sequences from *Ptr* and other related pathogens:

	Species	Accession #	Isolate
1	*Pyrenophora tritici-repentis*	MN052875	T176-3
2	*Pyrenophora tritici-repentis*	MN052876	T168-4
3	*Pyrenophora tritici-repentis*	MN052877	T25-4
4	*Pyrenophora tritici-repentis*	MN052878	T168-1
5	*Pyrenophora tritici-repentis*	MN052879	T168-7
6	*Pyrenophora tritici-repentis*	MN052880	T168-2
7	*Pyrenophora tritici-repentis*	MN052881	T25-7
8	*Pyrenophora tritici-repentis*	MN052882	T178-2
9	*Pyrenophora tritici-repentis*	MN052883	T178-1
10	*Pyrenophora tritici-repentis*	MN052884	T176-2
11	*Pyrenophora tritici-repentis*	MN052885	T17-2
12	*Pyrenophora tritici-repentis*	MN052886	T173-9
13	*Pyrenophora tritici-repentis*	MN052887	T44-1
14	*Pyrenophora tritici-repentis*	MN052888	T44-4
15	*Pyrenophora tritici-repentis*	MN052889	T168-3
16	*Pyrenophora tritici-repentis*	MN052890	T132-2
17	*Pyrenophora tritici-repentis*	MN052891	T176-1
18	*Pyrenophora tritici-repentis*	MN052892	T103-2
19	*Pyrenophora tritici-repentis*	MN052893	T75-1
20	*Pyrenophora tritici-repentis*	MN052894	T173-5
21	*Pyrenophora tritici-repentis*	MN052895	T168-8
22	*Pyrenophora tritici-repentis*	MN052896	T103-1
23	*Pyrenophora tritici-repentis*	MH017419	NZ1
24	*Pyrenophora tritici-repentis*	MH017416	SN002B
25	*Pyrenophora tritici-repentis*	MH017414	CC142
26	*Pyrenophora tritici-repentis*	MH017417	EW4_4
27	*Pyrenophora tritici-repentis*	MN062685	AB88-2
28	*Pyrenophora tritici-repentis*	HM234155	PTR-01
29	*Pyrenophora tritici-repentis*	MVBI01000015	5213
30	*Pyrenophora tritici-repentis*	AF004369	Pt-lc – H15
31	*Pyrenophora tritici-repentis*	U79662	86-124 – H16
32	*Pyrenophora tritici-repentis*		H14
33	*Pyrenophora teres f. maculata*	HM234165	H611
34	*Pyrenophora teres f. maculata*	HM234166	H312
35	*Pyrenophora teres f. maculata*	HM234167	H614
36	*Pyrenophora teres f. teres*	HM234162	H603
37	*Pyrenophora teres f. teres*	HM234163	H606
38	*Pyrenophora teres f. teres*	HM234164	H607
39	Phaeosphaeria nodorum	HM191251	Sn4
40	*Parastagonospora nodorum*	MH511822	WAC13666
41	*Parastagonospora nodorum*	MH511823	Fr15-02
42	*Bipolaris sorokiniana*	KX816409	WAI2674
43	*Phaeosphaeria nodorum*	EF108451	Sn01Aus.A1

44	*Phaeosphaeria nodorum*	EF108452	Sn01AUS.A2
45	*Phaeosphaeria nodorum*	EF108453	Sn01AUS.B2
46	*Phaeosphaeria nodorum*	EF108454	NNDKXE02-1
47	*Phaeosphaeria nodorum*	EF108455	Sn95SA.103
48	*Phaeosphaeria nodorum*	EF108456	SnSA95.113
49	*Phaeosphaeria nodorum*	EF108457	SnSA95.134
50	*Phaeosphaeria nodorum*	EF108458	SnSa95.8
51	*Phaeosphaeria nodorum*	EF108459	SnSA95.23
52	*Phaeosphaeria nodorum*	EF108460	SnKZ3-1-6
53	*Phaeosphaeria nodorum*	EF108461	SnCA1-3
54	*Phaeosphaeria nodorum*	EF108462	SnKZ30-5
55	*Phaeosphaeria nodorum*	EF108463	SnTJ1-3
