# Supplementary material for: Pyrenophora tritici–repentis in Tunisia: Race Structure and Effector Genes
Source: Front Plant Sci. 2019 Dec 18;10:1562. doi: 10.3389/fpls.2019.01562 (PMC6930679; doi:10.3389/fpls.2019.01562)
Supplement: Supplementary file 2 [file Table_2.pdf]

Supplementary table 2: Distribution of *Ptr* races (recovered from wheat) around the world

Geographical location	Countries	Total number of isolates	Races	Number of isolate per race	%	References
North America	Canada, USA, Mexico	1569	1	984	62.71	Aboukhaddour et al., 2013; Abdullah et al., 2017b; Ali et al., 1999; Ali and Francl, 2003; Ali et al., 2010; Engle et al., 2006; Lamari and Bernier, 1989b; Lamari et al., 1998; Lepoint et al., 2010; Leisova-Svobodova et al., 2010; MacLean et al., 2017; Singh et al., 2007; Freile, 2018
			2	450	28.68	
			3	57	3.63	
			4	44	2.8	
			5	14	0.89	
			Atypical	20	1.27	
South America	Argentina, Brazil, Uruguay	482	1	218	45.22	Ali and Francl, 2002; Bertagnolli et al., 2019; Gamba et al., 2012; Lepoint et al., 2010; Leisova-Svobodova et al., 2010, Moreno et al., 2015; Santana et al., 2008; Shaukat and Francl, 2002
			2	179	37.13	
			3	3	0.62	
			4	15	3.11	
			5	1	0.2	
			6	1	0.2	
			8	13	2.69	
			Atypical	52	10.78	
Europe	Finland, Romania, Lithuania, Latvia, Belgium, Luxembourg, Czech republic, Slovak republic	361	1	219	60.66	Abdullah et al., 2017a; Lepoint et al., 2010; Leisova-Svobodova et al., 2010; Mironenko et al., 2019**; Sarova et al., 2005
			2	19	5.26	
			3	27	7.47	
			4	29	8.03	
			6	3	0.83	
			8	10	2.77	
			Atypical	54	14.95	
North Africa	Tunisia , Algeria, Morocco	305	1	33	10.81	Benslimane et al., 2011; Gamba et al., 2017; Lamari et al., 1995; Lepoint et al., 2010; Strelkov et al., 2002; and this current study
			2	1	0.32	
			4	2	0.65	
			5	109	35.73	
			6	63	20.65	
			7	59	19.34	
			8	1	0.32	
			Atypical	37	12.13	
		153	1	92	60.13	

Around Caucasian Regions	North Caucasian regions, Fertile Crescent, Turkey, Georgia, Azerbaijan, Armenia, Syria		2	6	3.92	Lamari et al., 2005b; Lepoint et al., 2010
			3	8	5.22	
			5	18	11.76	
			7	13	8.49	
			8	16	10.45	
North Asia	Kazakhstan, Kyrghyzstan, Uzbekistan, Tajikistan, Russia, Siberia	450	1	276	61.33	Gultyaeva et al., 2018 ; Lamari et al., 2005; Leisova-Svobodova et al., 2010; Lepoint et al., 2010; Maraite et al., 2006; Mironenko et al., 2019**
			2	60	13.33	
			3	31	6.88	
			4	47	10.44	
			5	2	0.44	
			7	8	1.77	
			8	26	5.77	
South Asia	Pakistan, Nepal, India, Bangladesh, Iran	70	1	51	72.85	Ali et al., 2001; Lepoint et al., 2010; Momeni et al., 2014
			2	15	21.42	
			8	1	1.42	
			Atypical	3	4.28	
Australia	Australia, New Zealand	131	1/2	-	-	Antoni et al., 2010*; Weith, 2015*

* Based on PCR: the *ToxA* gene was present and the *ToxB* gene was absent

** Frequency relied on phenotypical data as PCR was not in agreement with phenotypes
